# Early neurological deterioration in cardiogenic cerebral embolism due to nonvalvular atrial fibrillation: Predisposing factors and clinical implications

**DOI:** 10.1002/brb3.1985

**Published:** 2020-12-04

**Authors:** Lin Cong, Weining Ma

**Affiliations:** ^1^ Department of Neurology Shengjing Hospital of China Medical University Shenyang China; ^2^ Department of Neurosurgery Shengjing Hospital of China Medical University Shenyang China

**Keywords:** acute ischemic stroke, biomarker, cardiogenic cerebral embolism, early neurological deterioration, hemorrhage transformation

## Abstract

**Purpose:**

The aim of the study was to investigate factors which may predispose patients to early neurological deterioration (END) and explore peripheral biomarkers for the prediction of END in cardiogenic cerebral embolism (CCE) patients.

**Methods:**

Patients diagnosed with CCE within 24 hr of onset between January 2017 and January 2019 were included in this study. END was defined as an increase of ≥2 on the National Institutes of Health Stroke Scale (NIHSS) or the emergence of new neurological symptoms within 3 days of admission. Binary logistic regression was used to investigate the factors associated with END. Receiver operating characteristic (ROC) curves were then generated to determine the predictive value of the potential biomarkers and the optimal cutoff values.

**Results:**

Of the 129 (male, 55.81%; mean age 71.85 ± 11.99 years) CCE patients, 55 patients with END were identified. Hemorrhage transformation (HT), coronary heart disease (CHD), diastolic blood pressure, cystatin C levels, NIHSS score, and platelet‐to‐lymphocyte ratio (PLR) at admission were independently associated with END. A peripheral cystatin C level ≥ 1.41 mg/L and a PLR ≥ 132.97 were predictive factors for END in CCE patients. The lymphocyte‐to‐monocyte ratio (LMR) was negatively independently associated with HT, and LMR < 2.31 may predict the occurrence of HT in patients with CCE.

**Conclusions:**

Of the potential predisposing factors considered, increased cystatin C and PLR were associated with END within 3 days of CCE, and a decreased LMR may have predictive value for HT in CCE patients.

## Graphical abstract

Early neurological deterioration (END) in the early phase of acute ischemic stroke (AIS) generally leads to a marked increase in poor prognosis due to diverse mechanisms. Cystatin C levels (cutoff value 1.41 mg/L, AUC 0.635, 95% CI 0.537–0.733, *p* = .009) and PLR (cutoff value 132.97, AUC 0.619, 95% CI 0.518–0.719, *p* = .02) were predictive of END, with high specificity and sensitivity. Hemorrhage transformation (HT) was independently associated with END, the lymphocyte‐to‐monocyte ratio (LMR) was negatively independently associated with HT, and LMR < 2.31 may predict the occurrence of HT in patients with cardiogenic cerebral embolism (CCE).

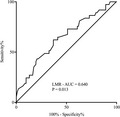

## 1 INTRODUCTION

Cardiogenic cerebral embolism (CCE) accounts for 15%–20% of ischemic strokes (IS). Atrial fibrillation (AF), especially nonvalvular atrial fibrillation (NVAF), is the most common cause of the embolism, accounting for about 50% of cerebral embolisms (Doufekias et al., 2008). Yamanouchi et al. found that 28% of IS in autopsied elderly patients were CCE, 56% of which were caused by NVAF (Yamanouchi et al., 1997). AF is a common arrhythmia, afflicting about 2% of the world's population. In recent years, the incidence of AF has risen. It is expected that by 2050, the prevalence of AF will rise more than 2.5 times, possibly due to an aging population (Miyasaka et al., 2006). The average age of AF patients has also increased steadily, currently ranging from 75 to 85 years (Lip, 2013; Palacio & Hart, 2002). The mechanism underlying cerebral embolism in patients with AF is complex. The main mechanism of pathogenesis involves a change in the structure and function of the left atrial appendage (LAA), which leads to blood stasis and induction of LAA thrombosis(Yamaji et al., 2002). The embolus then falls off and embolizes the intracranial artery. In CCE, the embolus is often large and causes sudden occlusion of the large and medium intracranial vessels (Hong et al., 2013).

In our previous studies, we have found that patients with CCE often have accompanying early neurological deterioration (END). END in the early phase of acute ischemic stroke (AIS) generally leads to a marked increase in poor prognosis due to diverse mechanisms (Alawneh et al., 2009). However, the predisposing factors associated with END in AIS patients have not yet been fully elucidated and no unified conclusions have been drawn. Previous studies have suggested that many factors may be associated with END, including systolic blood pressure, neurological functional deficits at admission, hyperglycemia, fibrinogen, and hemorrhage transformation (HT; Chung et al., 2015; Dziedzic, 2008; Seners et al., 2014). Nevertheless, studies on the predisposing factors for END in CCE patients are quite rare. Studies have shown that the incidence of HT in AIS is about 4%–7%, and HT is associated with END (Seet & Rabinstein, 2012; Seners et al., 2015). However, the incidence of and predisposing factors for HT in patients with CCE are also unclear. The aim of this study was (a) to investigate the incidence of and predisposing factors for END; (b) to identify the incidence and predisposing factors for HT; and (c) to examine possible peripheral biomarkers for the prediction of END and HT in CCE patients.

## 2 METHODS

### 2.1 Study population

From January 2017 to January 2019, 129 consecutive patients with CCE were enrolled in the study at the Department of Neurology and Emergency Department of Shengjing Hospital affiliated with China Medical University. The inclusion criteria were as follows: (a) acute onset, confirmed by cranial CT or MRI, with AIS, and in line with the diagnostic criteria for CCE: Patients may be caused by emboli in the heart. (1) At least one related cardiac source of emboli must be determined; (2) clinical and neuroimaging findings are similar to LAA ischemic stroke; and (3) previous transient ischemic attack (TIA) or stroke in more than one vascular area or evidence of systemic embolism supports the clinical diagnosis of cardiogenic stroke (Quan et al., 2020; Sacco et al., 2013; “Stroke‐‐1989. Recommendations on stroke prevention, diagnosis, and therapy. Report of the WHO Task Force on Stroke and other Cerebrovascular Disorders,” 1989); (b) history of AF or confirmed during admission according to an electrocardiogram or dynamic electrocardiogram; and (c) age > 18 years old. The exclusion criteria were as follows: (a) patients with a severe consciousness disorder (Glasgow Coma Scale < 9) who could not cooperate with the examination; (b) echocardiography suggesting valvular heart disease or congenital heart disease; or (c) other serious systemic diseases such as infection, heart and lung failure, malignant tumor, or liver and kidney dysfunction. Ethical approval was obtained from the Shengjing hospital of China Medical University Ethics Committee before beginning the study.

### 2.2 Data collection

Demographic variables, traditional risk factors, and routine laboratory test results were collected using the hospital's electronic medical record system. All patients underwent MRI within 48 hr of admission. MRI examinations were performed with an Achieva 3.0 Tesla scanner (Philips Healthcare). The MRI protocol included T_2_‐weighted imaging (T_2_WI), T_1_‐weighted imaging (T_1_WI), fluid‐attenuated inversion recovery imaging (FLAIR), diffusion‐weighted imaging (DWI), and time‐of‐flight MRA. HT was defined as the presence of intracranial hemorrhage in the initial ischemic region, confirmed by CT or MRI (Hacke et al., 1998). The abnormal area of DWI was manually delineated on the MR sequence. In order to calculate the total DWI lesion volume of each patient, the DWI abnormal area was added and multiplied by the section thickness (mm) and the intersection gap (mm). Two neurologists, who were blinded to the clinical data, interpreted all the neuroimaging studies and reached consensus.

All patients in our study had a history of AF or were recently diagnosed with AF after admission. The existence of a history of AF indicated that the patient was clearly diagnosed by a cardiologist and had been received medication or ablation of AF before hospitalization prior to hospitalization in this study. The recent diagnosis of AF indicated that patients with no history of AF were diagnosed by a neurologist at admission based on electrocardiogram or Holter electrocardiogram (Kirchhof et al., 2016).

### 2.3 Definition of END

Stroke severity was evaluated according to the NIHSS upon admission and at 24 hr postadmission, and both assessments were performed by the same neurologist (Lee et al., 2017). END was defined as follows: (a) an increase of more than 2 points in the total NIHSS score compared to the score at admission; (b) an increase of more than 1 point in the NIHSS specificity subitems, namely level of consciousness (1a–1c) or motor capacity (5a–6b); or (c) new neurological deficits despite no change in the NIHSS score (Seners et al., 2014).

### 2.4 Treatment

According to the guidelines of the European Heart Association and the European Stroke Society in 2016, anticoagulant therapy was initiated 3 days after stroke onset, and anticoagulant therapy was initiated 2 weeks after onset in patients with severe stroke and a high risk of HT (Kirchhof et al., 2017). Warfarin was given at a dose of 2.5 mg/day, and prothrombin time (PT) and international normalized ratio (INR) were reexamined after 3 days of anticoagulation therapy. The warfarin dosage was then adjusted according to the INR results and was increased or decreased 0.625–1.25 mg each time until the INR was controlled at 2–3 (Tse et al., 2013).

### 2.5 Statistical analysis

The Mann–Whitney *U* test or Student's *t* test was used to compare non‐normally or normally distributed variables, respectively. Continuous variables are presented as mean ± standard deviation if normally distributed; otherwise, the median interquartile range (IQR) is presented. The categorical variables are expressed in terms of frequency and percentage, and the data were compared using a chi‐square test. The independent variables were analyzed by logistic regression analysis. The sensitivity and specificity of significant variables and the optimal cutoff values for predicting END and HT in CCE patients were determined by receiver operating characteristic (ROC) curves. Statistical analysis was performed using SPSS 22.0 software (SPSS Inc.). For all the statistics, *p* values below .05 were considered significant.

## 3 RESULTS

### 3.1 Sample characteristics

In total, 129 patients met the inclusion criteria and were included in this study. The patients had a mean age of 71.85 ± 11.99 years, and 55.81% were male. None of the enrolled patients received intravenous tPA (IVT) and endovascular treatment (EVT). The baseline clinical data and laboratory results are shown in Table 1.

**TABLE 1:** Demographic, clinical, and laboratory data in cardiogenic embolism patients with and without END

	All (*n* = 129)	With END (*n* = 55)	Without END (*n* = 74)	*p* value
Man, *n* (%)	72 (55.81)	26 (47.27)	46 (62.16)	.092
Age, (years, mean ± *SD*)	71.85 ± 11.99	74.96 ± 12.30	69.53 ± 11.28	.01
Smoking, *n* (%)	47 (36.43)	16 (29.09)	31 (41.89)	.135
Alcohol drinking, *n* (%)	41 (31.78)	15 (27.27)	26 (35.14)	.343
Hypertension, *n* (%)	83 (64.34)	41 (74.55)	42 (56.76)	.037
Diabetes mellitus, *n* (%)	35 (27.13)	9 (16.36)	26 (35.14)	.018
Hyperlipemia, *n* (%)	29 (22.48)	15 (27.27)	14 (18.92)	.261
CHD, *n* (%)	27 (20.93)	20 (36.36)	7 (9.46)	<.001
History of stroke, *n* (%)	32 (24.81)	21 (38.18)	11 (14.86)	.002
HT, *n* (%)	37 (28.68)	23 (41.82)	14 (18.92)	.004
Epilepsy, *n* (%)	4 (3.1)	4 (7.27)	0 (0)	.018
NIHSS at admission	6.90 ± 6.24	9.58 ± 6.47	4.91 ± 5.27	<.001
Systolic blood pressure (mmHg)	146.39 ± 23.40	150.25 ± 23.75	143.51 ± 22.88	.106
Diastolic blood pressure (mmHg)	86.47 ± 13.59	89.73 ± 12.57	84.05 ± 13.89	.018
Infarct volume (mm^3^)	39.45 ± 77.80	50.15 ± 81.07	31.59 ± 74.90	.189
FBG (mmol/L)	7.04 ± 4.35	7.34 ± 5.53	6.82 ± 3.23	.504
Cystatin C (mg/L)	1.42 ± 0.62	1.61 ± 0.78	1.29 ± 0.41	.003
Urea (mmol/L)	5.64 ± 2.58	5.99 ± 3.14	5.39 ± 2.06	.194
Creatinine (umol/L)	81.20 ± 38.72	90.66 ± 53.09	74.16 ± 20.63	.016
Triglyceride (mmol/L)	1.05 ± 0.51	0.96 ± 0.51	1.12 ± 0.50	.069
Total cholesterol (mmol/L)	4.17 ± 0.99	3.98 ± 0.97	4.31 ± 0.99	.065
HDL (mmol/L)	1.18 ± 0.36	1.15 ± 0.28	1.20 ± 0.40	.370
LDL (mmol/L)	2.64 ± 0.93	2.52 ± 0.96	2.73 ± 0.90	.192
Hcy	19.0 ± 7.47	19.86 ± 8.49	18.36 ± 6.59	.262
HbAlc (%)	6.35 ± 1.64	6.32 ± 1.58	6.36 ± 1.70	.891
White blood cell count (10^9^/L)	8.59 ± 4.15	9.26 ± 5.25	8.10 ± 3.05	.116
Neutrophil count (10^9^/L)	6.18 ± 3.99	7.11 ± 4.99	5.49 ± 2.90	.022
Lymphocyte count (10^9^/L)	1.52 ± 0.63	1.36 ± 0.61	1.65 ± 0.62	.008
MCV	94.24 ± 5.62	93.76 ± 4.56	94.59 ± 6.30	.412
MCH	30.85 ± 2.57	30.57 ± 2.78	31.06 ± 2.39	.286
RDW	14.03 ± 1.46	14.37 ± 1.66	13.77 ± 1.25	.021
Monocyte count (10^9^/L)	0.61 ± 0.30	0.64 ± 0.37	0.59 ± 0.24	.280
Platelet count (10^12^/L)	187.48 ± 91.31	201.56 ± 129.06	177.01 ± 45.12	.132
Mean platelet volume	10.41 ± 3.10	10.16 ± 3.04	10.59 ± 3.15	.434
Lymphocyte‐to‐monocyte ratio	2.82 ± 1.44	2.40 ± 1.27	3.13 ± 1.50	.004
Neutrophil‐to‐lymphocyte ratio	5.30 ± 4.89	6.83 ± 5.81	4.16 ± 3.73	.002
Platelet‐to‐lymphocyte ratio	153.92 ± 126.15	193.16 ± 171.76	124.75 ± 63.70	.002

Note

Figures in parentheses are percentages, unless indicated otherwise

Abbreviations: CHD, coronary heart disease; Cys, C cystatin C; END, early neurological deterioration; FBG, fasting blood glucose; HbAlc, glycosylated hemoglobin; Hcy, homocysteine; HDL, high density lipoprotein; HT, hemorrhagic transformation; LDL, Low density lipoprotein; MCH, mean corpuscular hemoglobin; MCV, mean corpuscular volume; NIHSS, National institute of Health Stroke Scale; RDW, red cell distribution width.

### 3.2 Demographic, clinical, and laboratory data in cardiogenic embolism patients with and without END

Among the patients, 55 (42.64%) showed END within 72 hr of onset. We found that the patients with END were older (74.96 ± 12.30 vs. 69.53 ± 11.28; *p* = .01) and had a higher incidence of hypertension (74.55% vs. 56.76%; *p* = .037), a lower incidence of diabetes mellitus (16.36% vs. 35.14%; *p* = .018), a higher incidence of CHD (36.36% vs. 9.46%; *p* < .001), a higher incidence of stroke history (38.18% vs. 14.86%; *p* = .002), a higher incidence of HT (41.82% vs. 18.92%; *p* = .004), a higher incidence of epilepsy (7.27% vs. 0; *p* = .018), a higher NIHSS score at admission (9.58 ± 6.47 vs. 4.91 ± 5.27; *p* < .001), a higher diastolic blood pressure at admission (89.73 ± 12.57 vs. 84.05 ± 13.89; *p* = .018), a higher level of cystatin C (1.61 ± 0.78 vs. 1.29 ± 0.41; *p* = .003), a higher level of creatinine (90.66 ± 53.09 vs. 74.16 ± 20.63; *p* = .016), a higher neutrophil count (7.11 ± 4.99 vs. 5.49 ± 2.90; *p* = .022), a lower lymphocyte count (1.36 ± 0.61 vs. 1.65 ± 0.62; *p* = .008), a higher red cell distribution width (14.37 ± 1.66 vs. 13.77 ± 1.25; *p* = .021), a lower lymphocyte‐to‐monocyte ratio (LMR, 2.40 ± 1.27 vs. 3.13 ± 1.50; *p* = .004), a higher neutrophil‐to‐lymphocyte ratio (NLR, 6.83 ± 5.81 vs. 4.16 ± 3.73; *p* = .002), and a higher platelet‐to‐lymphocyte ratio (PLR, 193.16 ± 171.76 vs. 124.75 ± 63.70; *p* = .002) (Table 1).

Binary regression analysis was used to investigate the independent factors associated with END, and we found that cystatin C (OR 3.92, 95% CI 1.41–10.86, *p* = .009), HT (OR 4.87, 95% CI 1.60–14.85, *p* = .005), CHD (OR 10.37, 95% CI 2.48–43.31, *p* = .001), diastolic blood pressure at admission (OR 1.05, 95% CI 1.01–1.09, *p* = .007), NIHSS at admission (OR 1.05, 95% CI 1.01–1.09, *p* < .001), and PLR (OR 1.01, 95% CI 1.00–1.02, *p* = .006) were independently associated with END in CCE patients (Table 2).

**TABLE 2:** Logistic regression analysis of factors associated with END

	OR	95% CI	*p* value
Cystatin C (mg/L)	3.92	1.41–10.86	.009
Hemorrhage transformation	4.87	1.60–14.85	.005
CHD	10.37	2.48–43.31	.001
Diastolic pressure at admission	1.05	1.01–1.09	.007
NIHSS at admission	1.20	1.09–1.33	<.001
Platelet‐to‐lymphocyte ratio	1.01	1.00–1.02	.006

Abbreviations: CHD, coronary heart disease; Cys C, cystatin C; END, early neurological deterioration; NIHSS, National institute of Health Stroke Scale.

**FIGURE 1:**
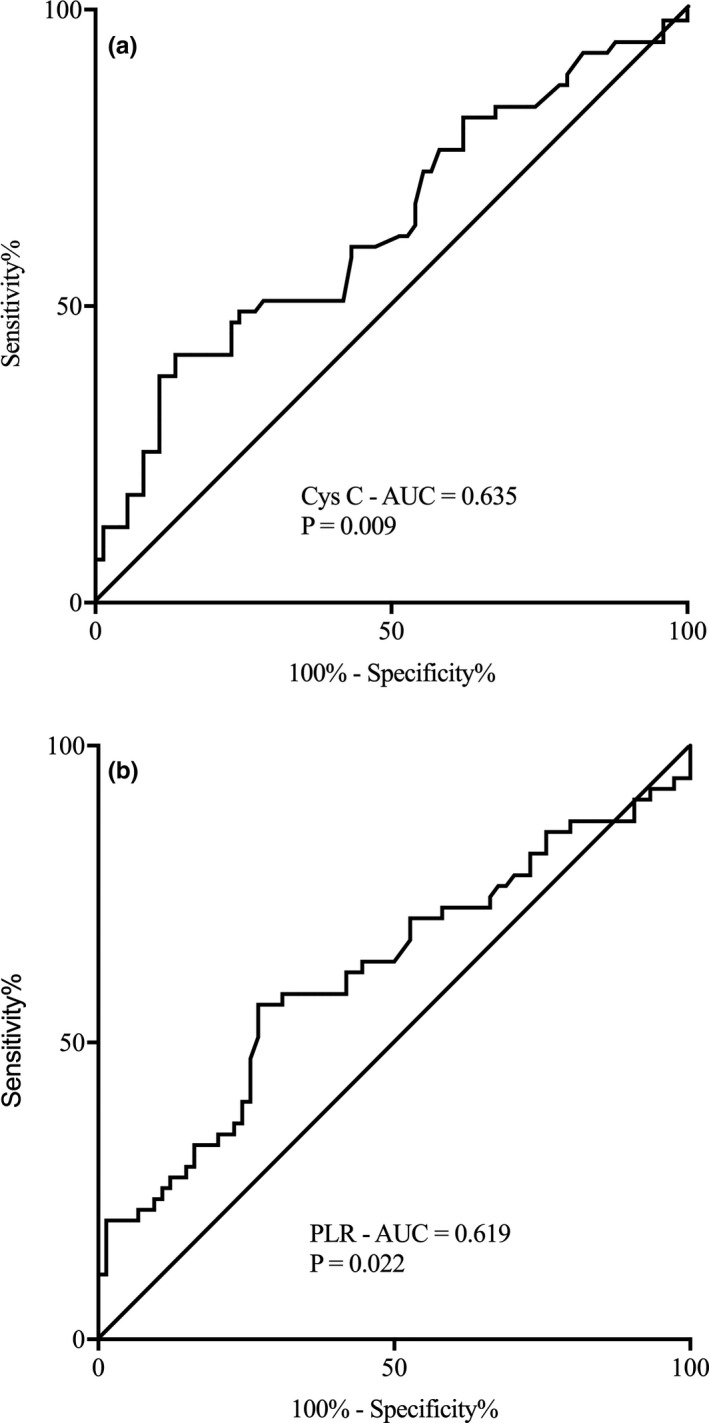
Receiver operating characteristic (ROC) curves for serum Cys C (a) and peripheral PLR (b) Cys C, cystatin C; AUC, area under the curve; END, early neurological deterioration; PLR, platelet‐to‐lymphocyte ratio; CCE, cardiogenic cerebral embolism

Next, ROC curve analysis was used to explore the potential value of peripheral biomarkers for the prediction of END. We found that cystatin C levels could predict END with a specificity of 75.7% and a sensitivity of 49.1% (cutoff value 1.41 mg/L, AUC 0.635, 95% CI 0.537–0.733, *p* = .009). PLR was also predictive of END, with a specificity of 73.0% and a sensitivity of 56.4% (cutoff value 132.97, AUC 0.619, 95% CI 0.518–0.719, *p* = .02) (Figure 1).

### 3.3 Demographic, clinical, and laboratory data in cardiogenic embolism patients with and without HT

Since HT is related to END in CCE patients, we determined which factors were associated with HT. We found that 37 (28.68%) patients had HT, and patients with HT had higher levels of cystatin C (1.60 ± 0.90 vs. 1.35 ± 0.45; *p* = .038), higher white blood cell counts (9.93 ± 5.50 vs. 5.61 ± 3.21; *p* = .010), higher neutrophil counts (7.60 ± 5.28 vs. 5.61 ± 3.21; *p* = .010), higher monocyte counts (0.71 ± 0.42 vs. 0.57 ± 0.23; *p* = .020), lower LMRs (2.35 ± 1.27 vs. 3.01 ± 1.47; *p* = .018), and higher NLRs (6.86 ± 5.76 vs. 4.67 ± 4.38; *p* = .021) (Table 3).

**TABLE 3:** Demographic, clinical, and laboratory data in cardiogenic embolism patients with and without HT

	With HT (*n* = 37)	Without HT (*n* = 92)	*p* value
Man, *n* (%)	19 (51.35)	53 (57.61)	.517
Age, (years, mean ± *SD*)	69.81 ± 13.57	72.66 ± 11.27	.223
Smoking, *n* (%)	11 (29.73)	36 (39.13)	.316
Alcohol drinking, *n* (%)	13 (35.14)	28 (30.43)	.604
Hypertension, *n* (%)	25 (67.57)	58 (63.04)	.628
Diabetes mellitus, *n* (%)	9 (24.32)	26 (28.26)	.649
Hyperlipemia, *n* (%)	6 (16.22)	23 (25.0)	.28
CHD, *n* (%)	8 (21.62)	19 (20.65)	.903
History of stroke, *n* (%)	8 (21.62)	24 (26.09)	.595
Epilepsy, *n* (%)	1 (2.70)	3 (3.26)	.869
NIHSS at admission	8.24 ± 6.27	6.34 ± 6.18	.121
Systolic pressure (mmHg)	149.27 ± 21.48	145.23 ± 24.15	.377
Diastolic pressure (mmHg)	87.08 ± 12.73	86.23 ± 13.98	.749
Infarct volume	59.92 ± 78.39	30.83 ± 76.36	.056
FBG (mmol/L)	7.11 ± 6.21	7.02 ± 3.37	.910
Cystatin C (mg/L)	1.60 ± 0.90	1.35 ± 0.45	.038
Urea (mmol/L)	5.49 ± 3.32	5.71 ± 2.24	.675
Creatinine (umol/L)	90.90 ± 58.40	77.30 ± 26.56	.071
Triglyceride (mmol/L)	1.05 ± 0.55	1.05 ± 0.49	.998
Total cholesterol (mmol/L)	4.09 ± 1.04	4.20 ± 0.98	.541
HDL (mmol/L)	1.22 ± 0.39	1.16 ± 0.34	.413
LDL (mmol/L)	2.58 ± 1.06	2.66 ± 0.87	.659
Hcy	19.38 ± 8.06	18.85 ± 7.26	.717
HbAlc (%)	6.58 ± 2.24	6.25 ± 1.33	.297
White blood cell count (10^9^/L)	9.93 ± 5.50	8.05 ± 3.36	.020
Neutrophil count (10^9^/L)	7.60 ± 5.28	5.61 ± 3.21	.010
Lymphocyte count (10^9^/L)	1.43 ± 0.65	1.56 ± 0.62	.287
MCV	94.97 ± 4.38	93.94 ± 6.04	.347
MCH	30.94 ± 3.21	30.82 ± 2.28	.802
RDW	14.24 ± 1.57	13.94 ± 1.41	.297
Monocyte count (10^9^/L)	0.71 ± 0.42	0.57 ± 0.23	.020
Platelet count (10^12^/L)	192.27 ± 115.16	185.55 ± 80.42	.707
Mean platelet volume	10.58 ± 3.17	10.34 ± 3.08	.687
Lymphocyte‐to‐monocyte ratio	2.35 ± 1.27	3.01 ± 1.47	.018
Neutrophil‐to‐lymphocyte ratio	6.86 ± 5.76	4.67 ± 4.38	.021
Platelet‐to‐lymphocyte ratio	172.41 ± 153.40	146.48 ± 113.49	.293

Note

Figures in parentheses are percentages, unless indicated otherwise.

Abbreviations: CHD, coronary heart disease; Cys C, cystatin C; END, early neurological deterioration; FBG, fasting blood glucose; HbAlc, glycosylated hemoglobin; Hcy, homocysteine; HDL, high density lipoprotein; HT, hemorrhagic transformation; LDL, Low density lipoprotein; MCH, mean corpuscular hemoglobin; MCV, mean corpuscular volume; NIHSS, National institute of Health Stroke Scale; RDW, red cell distribution width.

**FIGURE 2:**
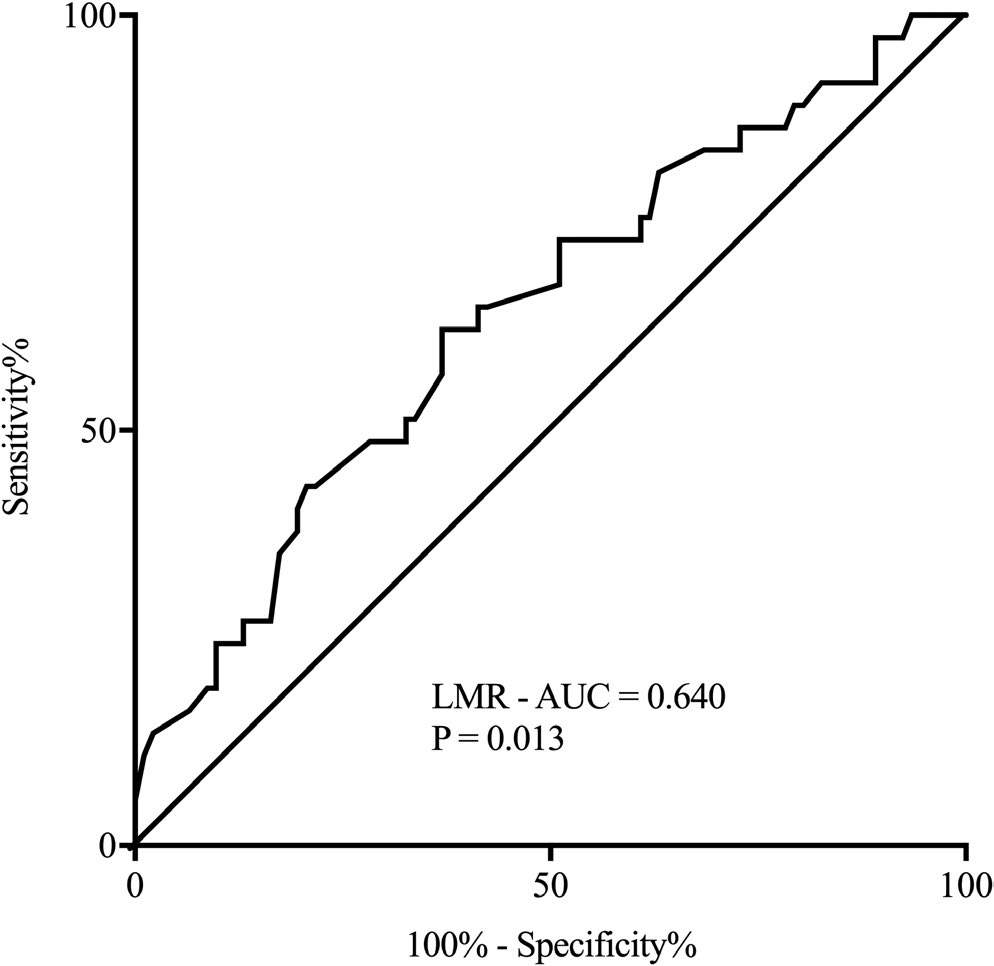
Receiver operating characteristic (ROC) curve for peripheral LMR. AUC, area under the curve; LMR, lymphocyte‐to‐monocyte ratio; CCE, cardiogenic cerebral embolism

Logistic regression showed that LMR (OR 0.69, 95% CI 0.49–0.95, *p* = .022) was negatively independently associated with HT in the CCE patients (table not shown). Moreover, LMR could predict HT with a specificity of 62.2% and a sensitivity of 63.0% (cutoff value 2.31, AUC 0.640, 95% CI 0.533–0.747, *p* = .013) (Figure 2).

## 4 DISCUSSION

In this study, we first determined the incidence of END in CCE patients and found that HT, CHD, diastolic blood pressure, NIHSS scores, cystatin C levels, and PLR at admission were independently associated with END. Furthermore, we found that cystatin C levels and PLR had value in predicting END. We also investigated factors related to HT in CCE patients and confirmed that LMR was negatively associated with HT and could also predict HT in CCE patients.

A large number of studies have confirmed that END is an independent risk factor for poor prognosis in stroke patients (Hui et al., 2018; Simonsen et al., 2016; You et al., 2019). Consequently, predictive factors for END are also of great interest. It has been reported that large artery atherosclerosis (Kim et al., 2016), initial glycemic variability (Hui et al., 2018), uric acid levels (Huang et al., 2018), mean platelet volumes (Oji et al., 2018), and fibrinogen levels (Lee et al., 2017) are associated with END. However, no unified conclusions regarding these risk factors have been reached, and no studies have been conducted on the predictive factors of END in CCE patients. Kim et al. recently reported that HT is an independent risk factor for END in AIS patients receiving endovascular thrombectomy (Kim et al., 2019). Gill et al. also found that severe HT was independently associated with worsening NIHSS scores in AIS patients receiving intravenous thrombolysis (Gill et al., 2016). These conclusions are consistent with our results. HT is independently associated with END, and the mechanism behind this correlation is complex. Usually, HT occurs in large artery atherosclerosis patients. Reperfusion injury may play a major role, and severe brain edema, damage to the blood–brain barrier, and excessive release of neurotoxic inflammatory mediators after HT may also be involved (Jeon et al., 2014; Juttler et al., 2014; Su et al., 2016). These patients also have higher NIHSS scores, which are consistent with our results. The incidence of AF in patients with CHD is significantly higher than that in healthy people, and CHD usually indicates more severe arteriosclerosis and is associated with poor prognosis in patients with AIS. Our results suggest that CHD is associated with END in patients with CCE (Drakopoulou et al., 2019; Ferrari & Fox, 2016; Wang et al., 2019). Blood pressure (BP) may contribute to the development of END and to poor outcomes by affecting cerebral perfusion (Toni et al., 1998). Other studies have also shown the importance of BP for prognosis after IS (Geeganage et al., 2011; Ko et al., 2010). Our study showed that increased diastolic blood pressure was associated with END in CCE patients, which is consistent with previous studies. Several studies have shown that cystatin C is associated with IS and could be considered a risk factor for AIS (Wang, Li, et al., 2019). Additionally, cystatin C is also independently associated with shorter survival in IS patients (Winovich et al., 2017). Kim et al. showed that cystatin C was a useful predictor of END in elderly patients with AIS (Kim et al., 2017). Our study confirmed that cystatin C is not only independently associated with END, but that cystatin C levels ≥ 1.41 mg/L can predict END in CCE patients. The PLR has been used as a convenient novel biomarker indicating inflammation, thrombosis, and plaque instability (Turkmen et al., 2013). Sung et al. reported that the PLR correlated with the severity of neurological impairment in patients after AIS (Sung et al., 2019). In addition, Zhang et al. recently reported that PLR could predict the severity of AIS and poor 30‐day outcomes (Zhang et al., 2019). In the present study, we further confirmed that PLR was independently related to END and identified the threshold value (PLR ≥ 132.97). PLR had a specificity of 73.0% and a sensitivity of 56.4% for predicting END. For END was a composite outcome that includes initial NIHSS, it was speculated he NIHSS was associated with END, and we did obtain that NIHSS was independently related to END at admission, which was consistent with the conclusion of Boulenoir et al. (2020).

As HT has not been reported to be associated with END in CCE patients, we further analyzed factors related to HT in this study. We found that LMR was negatively independently associated with HT, and LMR < 2.31 had a 63.0% sensitivity and 62.2% specificity for predicting HT. Ren et al. found that LMR on admission possessed good predictive value for AIS prognosis and that a lower LMR is closely related to AIS severity and poor prognosis (Ren et al., 2017). Park et al. reported that lower LMR on day 7 of AIS was associated with poorer prognosis at 3 months after stroke onset. LMR may be a useful biomarker for evaluating stroke‐induced immunosuppression (Park et al., 2018). Therefore, it is possible that decreased LMR may also suggest a poststroke inflammatory reaction in HT. Furthermore, this reaction may be involved in infarct evolution and may worsen the clinical outcome of patients with AIS.

The present study has a few limitations. First, it is a single‐center, retrospective study, and the sample size was small. Second, the PLR and LMR are dynamic indicators. We only recorded PLR and LMR at admission and did not remeasure them during the period of hospitalization. Third, we focused only on relatively early deterioration, and no long‐term follow‐up was conducted. Moreover, the sensitivity and specificity of cystatin C, PLR, or LMR are relatively low with all AUC less than 0.7, so their predictive values are of limited.

## 5 CONCLUSIONS

To the best of our knowledge, this is the first study to show that HT, CHD, diastolic blood pressure, cystatin C levels, NIHSS, and PLR at admission are independently associated with END. Furthermore, peripheral biomarkers, such as cystatin C and PLR, were predictive of END in CCE patients. LMR had a negative independent association with HT, and reduced LMR may have predictive value for HT. However, further studies with larger cohorts are needed to verify our findings.

## CONFLICT OF INTEREST

The authors declare that the research was conducted in the absence of any commercial or financial relationships that could be construed as a potential conflict of interest.

## AUTHOR CONTRIBUTIONS

Lin Cong collected clinical data, performed follow‐up of patients, completed statistical analysis, and wrote the manuscript. Weining Ma designed the study and contributed to editing the manuscript. All authors read and approved the final manuscript.

### PEER REVIEW

The peer review history for this article is available at https://publons.com/publon/10.1002/brb3.1985.

## Data Availability

The data of our study will be available via connecting with Dr. Weining Ma (corresponding author).
